# Signatures of cysteine oxidation on muscle structural and contractile proteins are associated with physical performance and muscle function in older adults: Study of Muscle, Mobility and Aging (SOMMA)

**DOI:** 10.1111/acel.14094

**Published:** 2024-02-08

**Authors:** Nicholas J. Day, Shane S. Kelly, Li‐Yung Lui, Tyler A. Mansfield, Matthew J. Gaffrey, Jesse B. Trejo, Tyler J. Sagendorf, Isaac K. Attah, Ronald J. Moore, Collin M. Douglas, Anne B. Newman, Stephen B. Kritchevsky, Philip A. Kramer, David J. Marcinek, Paul M. Coen, Bret H. Goodpaster, Russell T. Hepple, Peggy M. Cawthon, Vladislav A. Petyuk, Karyn A. Esser, Wei‐Jun Qian, Steven R. Cummings

**Affiliations:** ^1^ Biological Sciences Division Pacific Northwest National Laboratory Richland Washington USA; ^2^ San Francisco Coordinating Center California Pacific Medical Center Research Institute San Francisco California USA; ^3^ Department of Physiology and Aging University of Florida College of Medicine Gainesville Florida USA; ^4^ Department of Epidemiology University of Pittsburgh Pittsburgh Pennsylvania USA; ^5^ Department of Internal Medicine‐Gerontology and Geriatric Medicine Wake Forest University School of Medicine Winston‐Salem North Carolina USA; ^6^ Department of Radiology University of Washington Seattle Washington USA; ^7^ Translational Research Institute AdventHealth Orlando Florida USA; ^8^ Department of Physical Therapy University of Florida College of Medicine Gainesville Florida USA; ^9^ Department of Epidemiology and Biostatistics University of California San Francisco California USA

**Keywords:** cysteine oxidation, fitness, muscle, posttranslational modifications, power, redox

## Abstract

Oxidative stress is considered a contributor to declining muscle function and mobility during aging; however, the underlying molecular mechanisms remain poorly described. We hypothesized that greater levels of cysteine (Cys) oxidation on muscle proteins are associated with decreased measures of mobility. Herein, we applied a novel redox proteomics approach to measure reversible protein Cys oxidation in vastus lateralis muscle biopsies collected from 56 subjects in the Study of Muscle, Mobility and Aging (SOMMA), a community‐based cohort study of individuals aged 70 years and older. We tested whether levels of Cys oxidation on key muscle proteins involved in muscle structure and contraction were associated with muscle function (leg power and strength), walking speed, and fitness (VO_2_ peak on cardiopulmonary exercise testing) using linear regression models adjusted for age, sex, and body weight. Higher oxidation levels of select nebulin Cys sites were associated with lower VO_2_ peak, while greater oxidation of myomesin‐1, myomesin‐2, and nebulin Cys sites was associated with slower walking speed. Higher oxidation of Cys sites in key proteins such as myomesin‐2, alpha‐actinin‐2, and skeletal muscle alpha‐actin were associated with lower leg power and strength. We also observed an unexpected correlation (*R* = 0.48) between a higher oxidation level of eight Cys sites in alpha‐actinin‐3 and stronger leg power. Despite this observation, the results generally support the hypothesis that Cys oxidation of muscle proteins impairs muscle power and strength, walking speed, and cardiopulmonary fitness with aging.

## Graphical abstract

In this study, we applied a novel redox proteomics approach to quantitatively profile the levels of oxidation on protein cysteines in muscle tissue of older adults to identify signatures that may distinguish among varying degrees of mobility. In general, increased levels of Cys oxidation on key sarcomeric proteins were found to be negatively associated with muscle function, physical performance, and fitness.

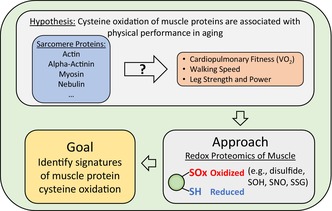

**Abbreviations**
ACTN2: alpha‐actinin‐2
ACTN3: alpha‐actinin‐3
ACTS: actin, alpha skeletal muscle
Cys: cysteine
LIMMA: linear models for microarray data
LN: liquid nitrogen
MYH7: myosin‐7
MYOM1: myomesin‐1
MYOM2: myomesin‐2
NEBU: nebulin
NOX: NADPH oxidases
OBSCN: obscurin
PCA: principal component analysis
PTM: post‐translational modification
ROS: reactive oxygen species
TMT: tandem mass tag
TNNI2: troponin I, fast skeletalmuscle
TPM2: tropomyosin beta chain
SOMMA: study of muscle, mobility and aging

## 1 INTRODUCTION

Aging is associated with diminished muscle and physical function which leads to mobility disability, loss of independence, and poorer quality of life (de Vries et al., 2012). However, the underlying molecular changes contributing to a decline in muscle function and physical performance remain unclear. In muscle, mitochondria and enzymes like NADPH oxidases (NOX) generate reactive oxygen species (ROS) such as superoxide radical (O_2_^•−^) and hydrogen peroxide (H_2_O_2_) (Bouviere et al., 2021). Studies have shown that ROS are important signaling molecules for underlying muscle function, including modulation of glucose transport (Henríquez‐Olguin et al., 2019), insulin sensitivity (Xirouchaki et al., 2021), and mitochondrial function (Kramer et al., 2015). Indeed, oxidants produced during exercise are essential for the exercise adaptive response, improving antioxidant capacity, and mitochondrial biogenesis (Powers et al., 2020). However, excessive or chronic exposure to ROS associated with aging causes fatigue, reduced contractile function of muscles, and blunted adaptive signaling (Andrade et al., 1998; Musci et al., 2019).

The free radical theory of aging first presented in 1956 proposed that tissue aging is driven by an accumulation of biomolecules that are damaged by free radicals (or ROS) created as by‐products of metabolic processes (Harman, 1956). The accrual of “damaged” biomolecules was thought to contribute to decline in physiological function as a causative factor of aging. This was followed by introduction of the “oxidative stress” concept in 1985 (and later updated in 2007), which defined “oxidative stress as the imbalance between oxidants and antioxidants in favor of the oxidants, leading to a disruption of redox signaling and control and/or molecular damage” (Sies, 1985). However, research in recent decades has prompted a conceptual shift, where ROS are critical for underlying biological processes and longevity, whereas ROS‐induced “oxidative damage” is now regarded as one of many factors in aging, rather than a singular cause (Sohal & Orr, 2012). As such, the original concept of ROS‐based “damage” is rather a potentially reversible process, involving “oxidative modifications” that alter mechanisms of physiological response and functional regulation, rather than causing dysfunction and degeneration. Consequently, the “redox stress” hypothesis has emerged as a theory to explain the relationship between ROS and aging, which posits that a shift to a more pro‐oxidizing redox state is a causal effect of the aging process (Sohal & Orr, 2012). At the molecular level, ROS‐mediated redox signaling can occur through thousands of occurrences of oxidative posttranslational modifications (PTMs) at the thiol group on protein cysteine (Cys) residues (Brandes et al., 2009). Several forms of these PTMs, such as S‐Glutathionylation, S‐Nitrosylation, S‐Sulfenylation, and disulfides, can be reduced back to a free thiol by antioxidant systems comprised of enzymes and small molecules, allowing them to function as reversible PTMs. Redox PTMs on Cys thiols modulate the activity, function, localization, and binding interactions of proteins, and their reversibility enables “switch”‐like behavior for transient signaling roles (Brandes et al., 2009). Reversible redox PTMs have been increasingly considered as a major regulatory mechanism in oxidative stress‐related diseases and aging (Kehm et al., 2021).

Mitochondrial dysfunction has been considered a hallmark of aging (López‐Otín et al., 2023). This situation enhances the production of ROS in aging tissues, resulting in a propensity towards an increasingly pro‐oxidizing redox state, leading to “redox stress”. The duality of ROS as both essential, but potentially detrimental for biological functions makes it of particular interest for studying the intersection of aging and muscle function, however identification of proteins that are modified by ROS in human aging muscle has not been investigated. The sarcomere is the fundamental contractile unit in muscle that is responsible for generating force. Research over the last 30 years has demonstrated that the force capacity of the sarcomere is an emergent property of all the proteins that contribute to the structure (Lehman et al., 2022). Consequently, changes or mutations in any one protein are sufficient to impact the function of the structure, such as those comprising the thick and thin filaments or the Z‐ and M‐lines.

We hypothesize that the levels of Cys oxidation on sarcomere proteins involved in muscle contraction and structure are associated with physical performance (walking speed), muscle function (leg strength and leg power), and cardiopulmonary fitness (VO_2_ peak). Previous studies have shown that Cys oxidation of sarcomere proteins can impact the function of the contractile unit. One example is based on the opposing effects of glutathionylation and nitrosylation on Cys134 of fast‐twitch troponin I, which functions within the troponin complex to modulate calcium activated force in muscle (Dutka et al., 2017). The large protein titin is also subject to oxidative modifications that modulate its mechanical properties such as elasticity and stiffness (Giganti et al., 2018). To test our hypothesis, we applied a novel redox proteomics approach to quantitatively profile the levels of oxidation on protein Cys in muscle tissue of older adults to identify signatures that may distinguish among varying degrees of physical performance.

## 2 RESULTS

### 2.1 Study cohort

**FIGURE 1:**
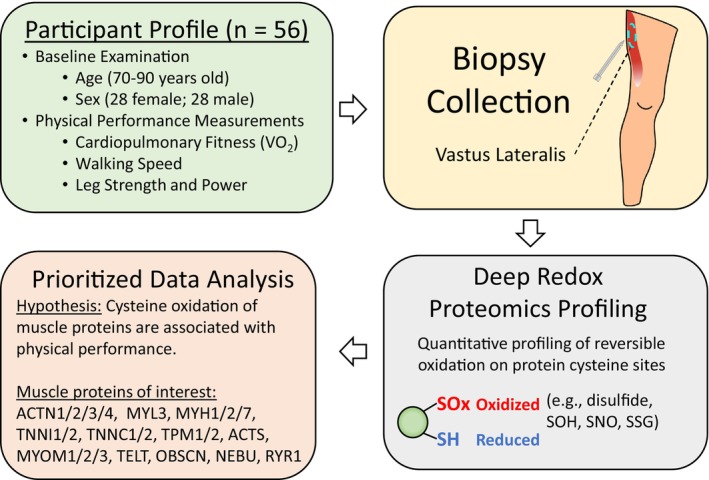
Study design and workflow. Participants undergo baseline physical examinations (age, height, weight, blood pressure, etc.) and measures of muscle function (leg strength and power), walking speed, and fitness (VO_2_ peak) as well as specimen collections (blood, urine, muscle, etc.) over multiple days (Cummings et al., 2023). Muscle biopsies were collected from vastus lateralis muscle. A deep redox proteomics profiling workflow was used to quantitatively measure Cys oxidation (i.e., all forms of reversible thiol PTMs). Analysis of redox proteomics data was prioritized for a select group of muscle contraction proteins (Uniprot ID format) to test the hypothesis that protein Cys oxidation is associated with functional measures.

The Study of Muscle, Mobility and Aging (SOMMA) was conceived to investigate how biological changes in muscle contribute to the decline in mobility—strength and walking—with aging. SOMMA recruited participants aged 70 years or older for extensive tests of physical performance (phenotypes) and collection of biospecimens (Cummings et al., 2023) (Figure 1). Needle biopsies from vastus lateralis muscle were collected for a variety of analyses, such as gene expression, mitochondrial respirometry (Mau et al., 2023), and protein Cys oxidation (this study). Participants in this exploratory study selected from the larger SOMMA cohort are representative of both sexes and age range (Figure S1A), as well as a range of phenotypes measured at the clinical sites. Participant characteristics of this study are summarized in Table 1, which were used for testing relationships between site‐specific Cys oxidation and phenotype data related to mobility.

**TABLE 1:** Participant characteristics (*n* = 56).

Characteristics	Group average
Age, years	77.6 ± 5.1
Sex	28 Men + 28 Women
Weight, kg	79.0 ± 18.8
VO_2_ peak, mL/min	1588.0 ± 498.1
Walking speed, m/s	1.0 ± 0.2
Leg strength (one repetition maximum, kg)	179.3 ± 67.2
Peak leg power, Watts	347.1 ± 185.8
All characteristics reported as mean ± *SD*	

### 2.2 Muscle redox proteome profiling

To test the hypothesis that protein Cys thiol oxidation on specific Cys residues of muscle proteins is associated with reduced muscle function and physical performance, we quantitatively profiled the redox proteome of muscle tissue from older adults. For reference, we define the term “Cys oxidation” as the total oxidation level of all reversible forms of PTMs on a given Cys residue, which serves as an overall measure of the redox state for a given Cys site (Zhang et al., 2021). Since the levels of oxidation on protein Cys residues are impacted by factors such as protein abundance, we devised a peptide‐level enrichment strategy that is adapted from our previously reported deep redox profiling workflow (Day et al., 2022). This workflow measures both global protein abundance and Cys oxidation (Figure 1; Figure S2A). We prioritized the analysis of a select group of 22 muscle structural and contractile proteins that are key components of sarcomeres to determine the association of Cys oxidation on these proteins with four measurements of physical performance (Figure 1; File S1). To conduct an initial comparison of Cys oxidation and protein abundance, we performed the first experiment using only 14 tissue samples. Statistical analysis using linear regression models adjusted for sex, age, and weight (see methods) was applied to test associations between protein Cys oxidation and muscle function (leg power and strength), walking speed, and fitness (VO_2_ peak). Due to the limited statistical power for the initial study, a criterion of *p* < 0.05 and adj. *p* < 0.25 was applied to identify statistically significant associations. The results revealed a greater number of significant associations for Cys oxidation (63 Cys sites) than protein abundance (nine proteins) (File S2). These initial experiment results confirm that redox proteome profiling of Cys oxidation provides more informative measurements for testing associations with muscle function than protein abundance. Thus, we focus on quantitative profiling of protein Cys oxidation in muscle tissues from 56 SOMMA participants (Table 1).

We used a tandem mass tag (TMT) multiplexed quantification strategy (Figure S2B), to allow for multiplexed quantification of Cys oxidation across 14 samples in each multiplex experiment. In total, we measured oxidation of 15,049 Cys sites, covering 4354 proteins. Using this dataset, we applied a 75% completeness threshold to analyze Cys sites with higher coverage of oxidation data across all plexes, yielding 7721 Cys sites from 2657 proteins. Principal component analysis (PCA) highlights the variation in protein oxidation profiles among participants, where most are dispersed throughout the plot with minimal separation due to variables such as sex (Figure S1B). We identified Cys oxidation at 173 sites corresponding to the 22 muscle structural and contractile proteins of interest (File S1), where the estimated average occupancy of oxidation for each site (Day et al., 2021) is reported in File S3. More than 70 Cys sites have estimated average occupancies over 20%, revealing many sites with high levels of modifications.

### 2.3 Statistical association analysis of Cys oxidation on key muscle proteins

Linear regression LIMMA models (Smyth, 2005) using sex, age, and weight as covariates were used to test associations, where the significance threshold was set at a *p* value < 0.05 (File S4). For each functional measurement, the levels of oxidation at significantly associated Cys sites (*p* < 0.05) were arranged by row‐wise hierarchical clustering and plotted as heatmaps, which revealed “signatures” (defined as clusters of Cys sites) with similar oxidation profiles (e.g., Figure S3). The statistical significance of each cluster in association with a given functional measure was further tested by LIMMA, where all clusters were statistically significant (adj. *p* < 0.05) (File S5). Cross‐correlational analysis of these clusters with the three other functional measurements was also performed, where more than 15 clusters showed correlation coefficients greater than ±0.3 for the intended phenotypes (File S5).

### 2.4 Signatures of Cys oxidation associated with functional measures

**FIGURE 2:**
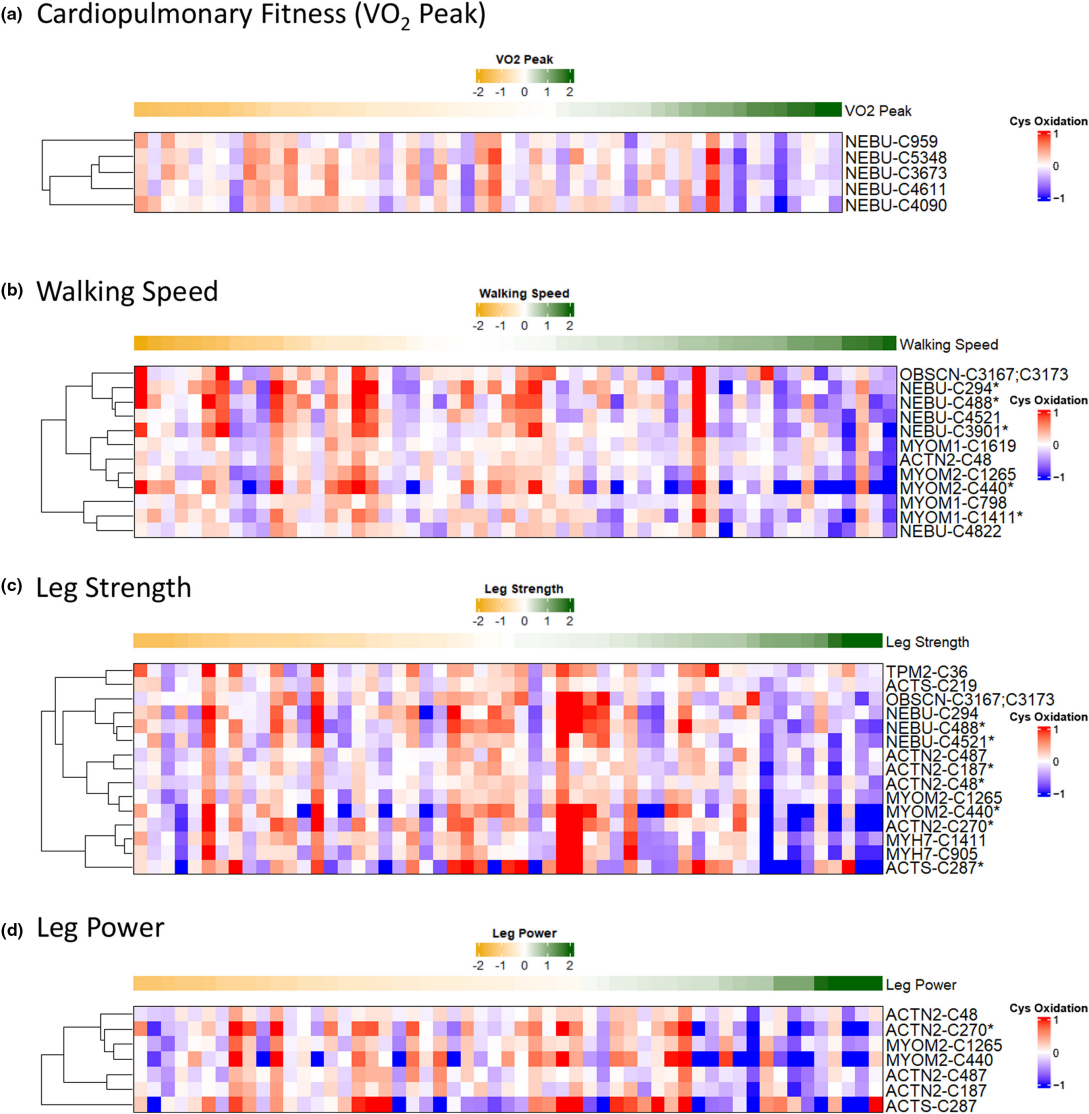
Selected clusters of Cys oxidation in significant association with fitness, muscle function, and physical performance. Cys sites on muscle proteins with oxidation levels significantly associated with a functional measure (File S4) were clustered and plotted in heatmaps (Figures S3, S5, S7, and S9). A representative cluster of Cys sites (also referred to as a “signature”) from each phenotype‐specific heatmap is shown in panels (a–d). Each row within the cluster represents a Cys site with oxidation levels that are significantly associated with a phenotype, while each column represents a participant. Columns are ranked in ascending order from left to right based on phenotypic measurement (i.e., lower to higher performance), where the phenotypic values are represented by median‐centered Z‐scores in the row above each cluster, while Cys oxidation levels were scaled by median‐centering in Log2 scale. Note that the row‐wise hierarchical clustering of Cys sites is reordered from the original corresponding clusters in Figures S3, S5, S7, and S9. Protein identities are in UniProt format. Sites passing an adjusted *p* < 0.05 cutoff are denoted by “*”. (a) Peak volume of oxygen consumption (VO_2_ peak; mL/min) as a measure of cardiopulmonary fitness. (b) Walking speed from a 400 m walking test (m/s). (c) Leg strength as measured by one repetition maximum (kg). (d) Leg power measured as the highest peak power (watts) generated.

**FIGURE 3:**
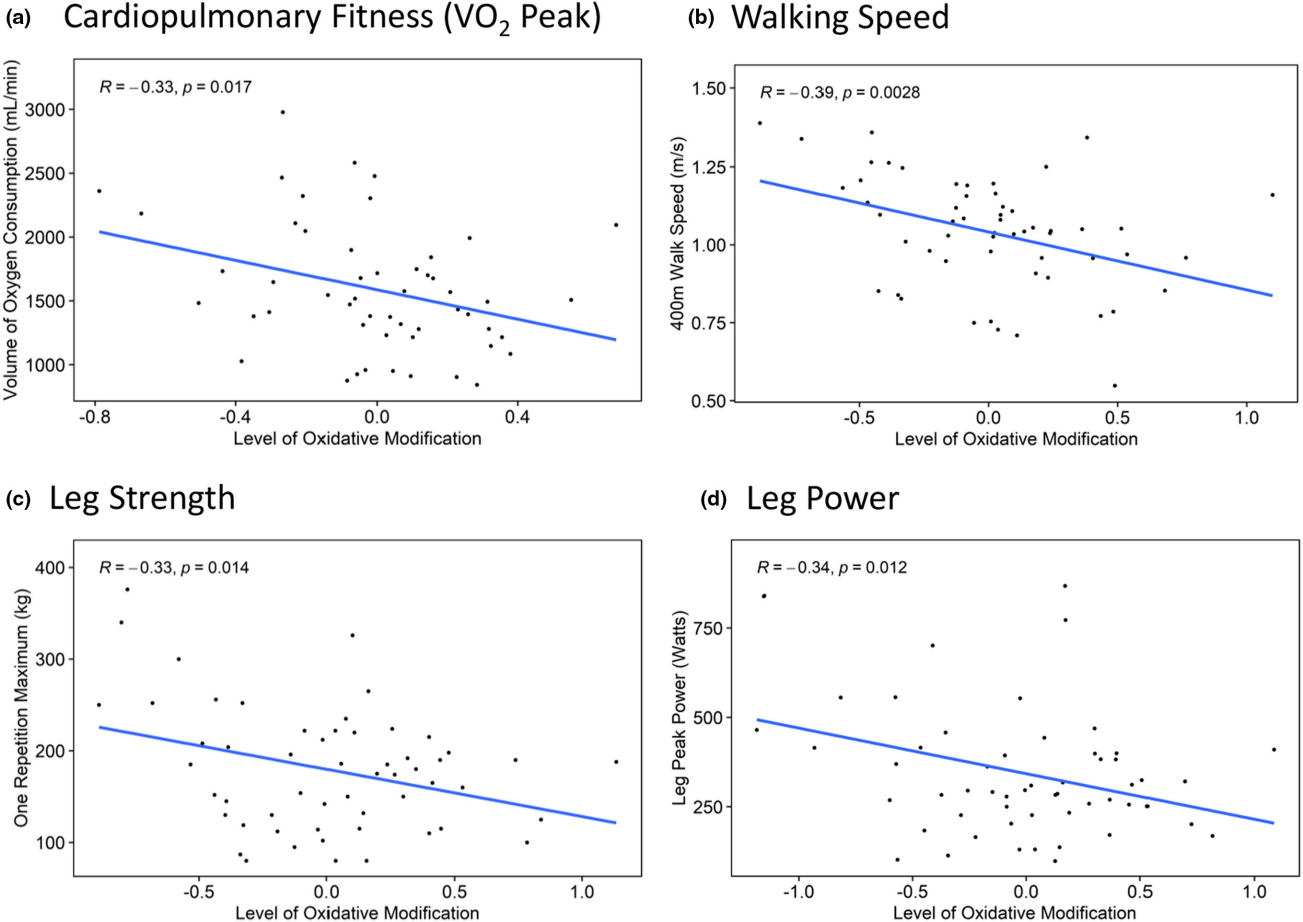
Selected clusters of Cys oxidation are negatively associated with fitness, muscle function, and physical performance in older adults. Clusters within each phenotype‐specific heatmap (Figures S3, S5, S7, and S9) were plotted for correlation analysis (Figures S4, S6, S8, and S10). Representative correlations of clusters highlighted in Figure 2 are shown in panels (a–d), where the mean level of oxidation (in Log2 scale) across all Cys sites in a cluster were plotted for each participant against their phenotypic measurement. *R* and *p* values are derived from Pearson correlation. (a–d) Representative Pearson correlations for (a) VO_2_ peak, (b) walking speed, (c) leg strength, and (d) leg power.

We first examined the clusters of Cys sites that show statistically significant associations with VO_2_ peak, which is considered the gold standard measure of cardiorespiratory fitness. A heatmap of all 19 Cys sites that passed our statistical significance cutoff (*p* < 0.05) revealed that oxidation levels on specific Cys sites of nebulin (NEBU), obscurin (OBSCN), and myomesin‐2 (MYOM2) were associated with cardiopulmonary fitness (Figure S3). Pearson correlation was used to further test the significance of the clusters with a given phenotype. The correlation plots of each cluster are shown in Figure S4, where clusters 2 and 4 showed a significant negative association (*R* < −0.30; *p* < 0.05). Among the clusters, one comprised of 5 NEBU Cys sites showed a predominantly negative association with VO_2_ peak (Figures 2a and Figure 3a), suggesting that the oxidation levels on specific Cys sites of NEBU are functionally related to overall cardiopulmonary fitness.

How quickly individuals complete a 400‐m walk is a key metric of mobility in older adults, thus we next tested associations between protein Cys oxidation and average walking speed. As shown in the overall heatmap (Figure **S5**), a total of 35 Cys sites were associated with walking speed, which include 14 out of the 22 proteins being tested (File S1). Correlation analysis (Figure S6) indicated that all clusters except cluster 5 displayed significant, negative associations with walking speed (*R* < −0.30; *p* < 0.05). Figure 2b shows a cluster of 12 Cys sites (i.e., cluster 2 of Figure S5) corresponding to myomesin‐1 (MYOM1), alpha‐actinin‐2 (ACTN2), MYOM2, NEBU, and OBSCN that were significantly associated with walking speed. The negative correlation (*R* = −0.39, *p* = 0.028; Figure 3b) suggested that there is a negative association between Cys oxidation of these proteins and walking speed.

We next studied the association between leg strength, leg power, and Cys oxidation. As shown in the heatmap (Figure S7), a total of 41 Cys sites from 15 proteins displayed different levels of association with leg strength. Correlation analysis of each cluster (Figure S8) revealed that only clusters 5 and 6 had significant negative associations (*R* < −0.30; *p* < 0.05). Figures 2c and 3c highlights the cluster of Cys sites (cluster 6 from Figure S7; *R* = −0.33, *p* = 0.014) from several proteins such as the alpha isoform of skeletal muscle actin (ACTS), myosin‐7 (MYH7: slow myosin heavy chain), tropomyosin beta chain (TPM2), ACTN2, OBSCN, NEBU, and MYOM2. Furthermore, Figure S9 shows 33 Cys sites from 11 proteins that were significantly associated with leg power. Clusters 2 and 5 (Figure S10) had either a significant negative or positive correlation with peak leg power (*R* < −0.30 or *R* > 0.45; *p* < 0.05). Figures 2d and 3d shows a cluster containing 7 Cys sites (cluster 2 of Figure S9) corresponding to ACTS, ACTN2, and MYOM2 with a negative association (*R* = −0.34; *p* = 0.012) between leg power and Cys oxidation.

**FIGURE 4:**
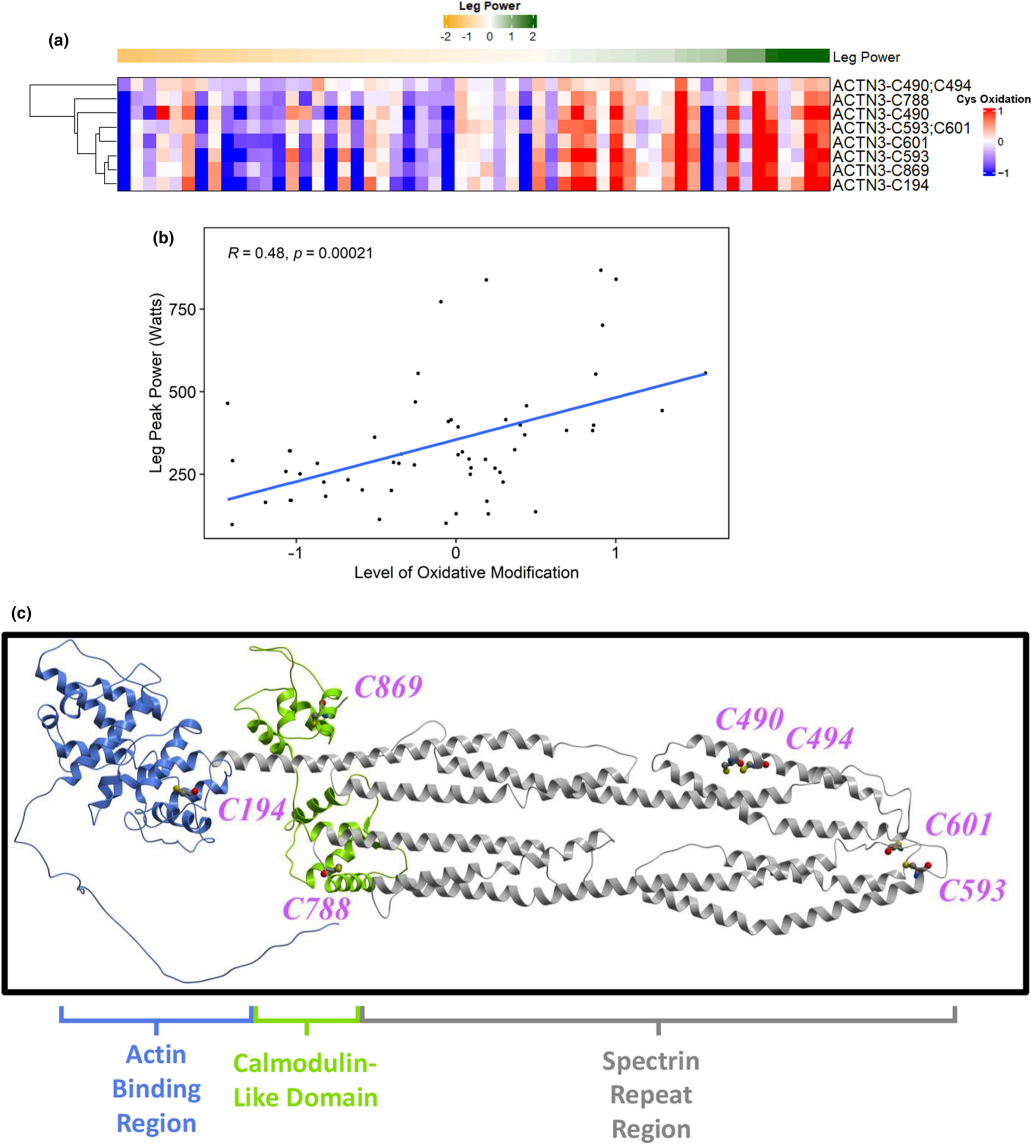
Unique, significant positive association between ACTN3 and leg power. (a) Representative heatmap of ACTN3 Cys sites identified as significantly associated with the leg power phenotype. Each row represents a Cys site with oxidation levels that are significantly associated with a phenotype, while each column represents a participant. Columns are ranked in ascending order from left to right based on phenotypic measurement (i.e., lower to higher performance), where the phenotypic values are represented by median‐centered Z‐scores in the row above the cluster, while Cys oxidation levels were scaled by median‐centering. Note that the row‐wise hierarchical clustering of Cys sites is reordered from the original corresponding cluster in Figure S9. Protein identities are in UniProt format. (b) Cys sites from the ACTN3 cluster in panel A were plotted for correlation analysis, where the mean level of oxidation across all Cys sites in a cluster were plotted for each participant against their phenotypic measurement. *R* and p values are derived from Pearson correlation. (c) AlphaFold‐predicted structure of ACTN3 with all significantly associated Cys sites denoted by magenta colored text. The Actin binding region (AA1‐262), neck and spectrin repeat region (AA263‐758), and calmodulin‐like domain containing two pairs of EF hands (AA759‐901) are colored in blue, gray, and green, respectively.

In agreement with our general hypothesis, most of the clusters exhibited a negative association between Cys oxidation and all functional measures. However, we observed a surprisingly different cluster for alpha‐actinin‐3 (ACTN3), which shows a significant positive correlation (*R* = 0.48, *p* = 0.00021; Figure 4a,b). This specific cluster contains 8 Cys sites that are exclusive to ACTN3, suggesting potential functional regulation of this protein involved in leg power. Interestingly, ACTN2 is a homolog of ACTN3 with roughly 80% identity but does not show a similar relationship between oxidation and leg power (Figure 2d). From a structural standpoint, these sites were mapped to the predicted structure of ACTN3, where we identified two pairs of Cys sites within the spectrin repeat region that are potentially in close enough proximity to form structural disulfides (Figure 4c, Cys490;494 and Cys593;601). Cys194 is found within the actin binding domain, while Cys788 and 869 are each found within a pair of EF hands that make up the Calmodulin‐like domain (Figure 4c), suggesting that redox regulation of Cys residues is a potential mechanism for modulating multiple functional regions of ACTN3.

## 3 DISCUSSION

Our current study not only provides a first‐time view into the molecular landscape of protein Cys oxidation in aging human muscle tissues, but also demonstrates that the levels of Cys oxidation on key muscle sarcomere proteins are generally negatively associated with poorer physical performance. In doing so, the data supports the hypothesis that Cys oxidation signatures are associated with muscle and physical performance. Linear regression and correlation analyses led to identification of clusters with Cys sites whose oxidation levels are significantly associated with muscle function (leg strength and power), physical performance (walking speed), and fitness (VO_2_ peak). These clusters contain several proteins that contribute to sarcomere structure as well as contractile activity such as NEBU, OBSCN, ACTS, MYH7, ACTN2, TPM2, MYOM1, and MYOM2.

For many decades, oxidative stress has been speculated to be a driver of muscle pathologies and aging, however this has not been previously tested in humans. Prior to this study, the relationship between Cys oxidation and aging has been tested in animal models (Campbell et al., 2019; Xiao et al., 2020), but not in humans. The findings presented in this study may begin to unravel the relationship between protein Cys oxidation and muscle function that is emerging as a regulatory mechanism in muscle biology. Given the importance of oxidative stress in the aging process, the application of redox proteomics has value for studying other domains in aging research to explore molecular mechanisms of aging and chronic aging‐related diseases.

### 3.1 Implications of Cys oxidation on muscle proteins

Among the structural and contractile proteins that were investigated, oxidation levels at Cys sites on several proteins repeatedly emerged as significantly associated with different measures of muscle function, physical performance, and fitness, emphasizing their potential importance in aging muscle and mobility. One such case is nebulin, where it was found in multiple clusters that were significantly and negatively associated with all four phenotypic measurements (File S4), along with its exclusive cluster for VO_2_ peak (Figure 2a). With a total of 6669 amino acids, nebulin is a giant protein that serves as an integral component of the skeletal muscle thin filament. It contributes to structure through regulation of thin filament length and plays a role in muscle contraction (Slick et al., 2023). Oxidation of Cys sites on this protein may disrupt these essential interactions and dysregulate the organization of actomyosin filaments. Interestingly, the NEBU cluster shown in Figure 2a shows a negative cross‐correlation (*R* < −0.30) with the three other phenotypes (File S5), suggesting that NEBU Cys oxidation is functionally important to all four measures of physical performance.

One of the most striking observations in this study revolves around the association of Cys oxidation and leg peak power for ACTN3, also known as the “gene of speed” (Figure 4a,b) (Pickering & Kiely, 2017). Contrary to our hypothesis and the notion that higher Cys oxidation results in a decline of muscle performance, the oxidation levels on multiple ACTN3 Cys sites were positively correlated with peak leg power (*R* = 0.48). Additionally, this ACTN3 cluster showed moderate positive cross‐correlation with VO_2_ peak (*R* > 0.30), while the ACTN2‐containing cluster showed moderate negative cross‐correlations with both walking speed and leg strength (*R* < −0.36) (File S5), emphasizing the importance of ACTN proteins in physical performance. Alpha‐actinin proteins bind and crosslink overlapping F‐actin filaments at the Z line in the sarcomere, making them important proteins for muscle integrity and quality (Burgoyne et al., 2015). Thus, oxidation in the different functional domains of ACTN3 may regulate its function in generating maximum muscular power. The calponin‐homology domains of alpha‐actinin are important for actin binding and conformational changes of the protein, where oxidation of Cys194 was observed (Haywood et al., 2016) (actin binding region; Figure 4c). Alpha‐actinin EF‐hand domains interact with titin, where we saw oxidation of Cys788 and Cys869 (Young & Gautel, 2000) (calmodulin‐like domain; Figure 4c). Disruption of this interaction impacts myofilament lattice spacing and cross‐bridge kinetics (Rodriguez Garcia et al., 2023). Additionally, Cys868 of ACTN3 (Cys869 in human) was identified as redox‐sensitive in an aging mouse study (McDonagh et al., 2014) (Figure 4a), supporting the notion that ACTN3 may be a redox‐regulated protein.

A number of factors could drive the highly contrasting alpha‐actinin 2 and 3 oxidation profiles, such as the ACTN3 genotype, where the distribution of individuals homozygous for a premature stop codon R577X polymorphism is estimated to be 20% (North et al., 1999). With this frequency, it is possible that some subjects in this study may have limited expression of ACTN3, which would affect the levels of protein Cys oxidation detected in our assay. The ACTN3 genotype may also influence muscle type I slow twitch/type II fast twitch fiber distribution (Vincent et al., 2007). While ACTN2 is expressed in both fiber types, the exclusive expression of ACTN3 in type II fast twitch muscle fibers also suggests fiber‐type specific physiological functions, which have differing responses to oxidants (Anderson & Neufer, 2006) and could help to explain this observation. Subsequent studies on the role of redox‐based PTMs on ACTN3 are warranted to gain a better understanding of this relationship and its functional regulation.

Besides clusters exclusive to single proteins, we also observed instances of oxidized Cys sites on proteins like TNNI2 and ACTS that have been previously reported in the literature. Our assay identified oxidation at Cys134 of fast‐twitch troponin I (TNNI2) as significantly associated with other clusters in the walking speed and leg power analyses (File S2; Figure S5, S7). Previous structural analysis work identified this Cys site as solvent‐accessible, suggesting it is oxidant‐accessible (Gross & Lehman, 2013). Other work revealed that the site undergoes S‐Glutathionylation and S‐Nitrosylation (Dutka et al., 2017) that modulate Ca^2+^ sensitivity and force generation, emphasizing this as a redox‐sensitive site. Another protein is skeletal muscle alpha actin, which is one of the basic components of the thin filaments in muscle but is also susceptible to Cys oxidation. We identified Cys219 on ACTS as significantly associated with leg strength (Figure 2c), while Cys287 was significantly associated with both leg strength and leg power (Figure 2c,d). These sites have been identified as redox‐sensitive Cys residues in prior work comparing adult and aged mouse skeletal muscle (McDonagh et al., 2014). Oxidation of actin can disrupt interactions with myosin, resulting in attenuated contraction and force production (Elkrief et al., 2022).

### 3.2 Limitations of this study

This study has several limitations to be acknowledged. While this study focused on measurements of Cys oxidation, global protein abundance profiling could provide additional insight into the biology of impaired muscle performance. Deep profiling of global protein abundances provides more resolution on other aspects, such as gene expression or protein degradation, which can help to interpret observations beyond the level of PTMs. With 56 community‐based volunteers, the study has limited statistical power to test associations and other confounding factors such as race, metabolism, and lifestyle, which may influence the heterogenous patterns of Cys oxidation. For example, we and others have previously demonstrated increased Cys oxidation in skeletal muscle after fatiguing contractions, suggesting significant physical activity in the day or hours prior to the biopsy could be confounding (Kramer et al., 2018; Pugh et al., 2021). For this work, visits were coordinated to avoid any physical activity on the same day as muscle biopsy collection, such as treadmill or strength testing, which could influence Cys oxidation. The SOMMA population is predominantly non‐Hispanic White, and given our sample size, we have limited ability to test whether associations between signatures of Cys thiol oxidation and our functional outcomes vary by race or ethnicity. The cross‐sectional design allows for testing the concurrent effects of muscle protein oxidation on muscle and physical performance, however repeated assessments are needed to test how Cys oxidation predicts change in performance with aging. Measurements of antioxidant capacity are also warranted, as the levels of antioxidant enzymes (e.g., mitochondrial superoxide dismutase, glutathione peroxidase, catalase) can vary among individuals (Picard et al., 2011). Indeed, a parallel SOMMA study revealed that elevated levels of gene expression for enzymes involved in countering oxidative stress were associated with better function of all phenotypes investigated in this study (see Tranah et al. in same issue). Nonetheless, this first study in older adults has several strengths. It has the power to detect significant associations of muscle protein oxidation with important components of mobility. The state‐of‐the‐art assays included an array of proteins known to play important roles in muscle functions and identified several pronounced signatures of Cys thiol oxidation that associate well with the measures of muscle function, physical performance, and fitness investigated in this study.

In summary, this study supports the hypothesis that higher levels of Cys oxidation on key muscle proteins are largely negatively associated with several measures of muscle function, physical performance, and fitness. These findings should be further tested in a more expanded study or a longitudinal study that also includes a younger group of participants to improve our understanding between Cys oxidation and age. Such work would help guide physiological studies needed to improve our understanding of the complex impact of Cys oxidation on muscle and physical function. This study also suggests that interventions that reduce the levels of oxidative modification of muscle proteins may improve or slow the loss of muscle function and fitness.

## 4 MATERIALS AND METHODS

### 4.1 Participants and cohort design

The Study of Muscle, Mobility and Aging (SOMMA; https://www.sommaonline.ucsf.edu) is a cohort study of 879 men and women aged 70 or older who were recruited at University of Pittsburgh and Wake Forest University School of Medicine. A description of the design and methods of SOMMA has been published (Cummings et al., 2023). Participants were eligible for this study if they were willing and able to complete a muscle tissue biopsy and magnetic resonance spectroscopy (MRS). All participants provided written and informed consent and the Western IRB‐Copernicus Group (WCG) Institutional Review Board approved the SOMMA (WCGIRB #20180764). For this exploratory redox proteomics profiling study, 56 participants were selected partially based on tissue sample availability and to capture a fair distribution across the cohort age range (Table 1).

### 4.2 Collection of clinical parameters and phenotype data

SOMMA baseline assessments were completed over the course of several days (Cummings et al., 2023). Age, race, and sex were assessed by questionnaire. Body weight was measured on a balance beam or digital scales. To determine usual walking speed, participants were instructed to walk 10 complete laps around a 40‐meter course at their usual pace and without overexerting themselves nor any assistive device other than a straight cane. The total time in seconds to walk 400 meters includes the rest time if the participant stopped walking during the test and was used to calculate walking speed in m/sec. Leg extension strength was assessed by a one repetition max (1RM) on a Keiser Air 420 exercise machine, and peak power was measured across the 40%–70% range of leg extension.

Cardiorespiratory fitness was assessed by a cardiopulmonary exercise test (CPET). Participants walked for 5 min at a preferred walking speed, and progressive symptom‐limited exercise protocol ensued with increases in speed (0.5 mph) and incline (2.5%) in 2‐min increments using a modified Balke protocol or a manual protocol. Cardiorespiratory fitness was determined as VO_2_ peak (mL/min), which was identified as the highest 30‐s average of VO_2_ (mL/min) achieved.

### 4.3 Muscle biopsy sample collection

Percutaneous biopsies were collected from the middle region of the vastus lateralis muscle under local anesthesia using a Bergstrom canula with suction (Evans et al., 1982). Following this, the specimen was blotted dry of blood and interstitial fluid and dissected free of any connective tissue and intermuscular fat. Samples were flash frozen in liquid nitrogen (LN) and stored in the vapor phase of LN. Samples were shipped to PNNL on dry ice for proteomics analysis.

### 4.4 Redox proteomics and mass spectrometry

Muscle biopsy samples (20–30 mg) were processed as previously described (Day et al., 2022). Two additional samples were generated by pooling tissue from the other samples to quantify both oxidized and reduced protein thiols (“total thiol”, Figure S2A). After acetone precipitation, the proteins were resuspended in a 200 mM Tris (pH 8); 8 M Urea buffer, reduced with DTT, and digested overnight with sequence‐grade trypsin (Promega) at a 1:50 (μg trypsin: μg protein) ratio. The peptides were cleaned up by C‐18 SPE and 100 μg of peptide from each sample was labeled with TMT18plex reagents (ThermoFisher; Figure S2B). 133C and 134 N were not used for quantification of total oxidation due to potential interference from the 134C and 135 N tags used to quantify total thiol. The labeled peptides were then resuspended in 250 mM HEPES pH 8, reduced with DTT at a final concentration of 5 mM, and a 100 μg aliquot of peptide was collected for global proteomics. The remaining peptide was diluted 6‐fold with 25 mM HEPES pH 7.7 to obtain a DTT concentration of ~0.8 mM to permit enrichment of Cys‐containing peptides with thiol affinity resin. The enrichment procedure is adapted from our RAC‐TMT workflow (Guo et al., 2014). After enrichment, the enriched redox peptides and global peptides were alkylated with iodoacetamide at 4 times the molarity of the DTT present in the samples and desalted using C‐18 SPE.

20 μg of enriched redox peptides were collected into 12 concatenated fractions as previously described (Day et al., 2022). Instrument runs were grouped by fraction order within each plex and arranged using a block design strategy (i.e., block 1 = fraction 1 from each plex) to avoid introduction of confounding factors due to decay of instrument performance. Conditions and parameters for LC–MS/MS runs are described elsewhere (Day et al., 2023). Samples of global peptide from each plex were prepared at a concentration of 0.1 μg/μL and used for single injections that were run on the same instrument as enriched redox peptides.

### 4.5 MS/MS data processing and statistical analysis

Raw data were searched against a database containing the Uniprot *Homo sapiens* proteome (release 2023_1 with 20, 407 entries) and common contaminants using MS‐GF+ with search parameters specified elsewhere (Day et al., 2022). TMT reporter ion intensities were extracted by MASIC as described in (Day et al., 2022). Data from all fractions of redox multiplexes were aggregated together to the site‐centric level and summarized using the R tool “PlexedPiper” (https://github.com/PNNL‐Comp‐Mass‐Spec/PlexedPiper). Additional processing steps are as follows. The redox data was corrected for changes in protein abundance by taking the median intensities of un‐normalized global peptide‐centric data and scaling them to the average median intensity of the redox data, which was then used to center corresponding channels in the redox data. Data were corrected for multiplex‐to‐multiplex batch effects as well as biopsy collection site using the “ComBat” function in the R package “sva” (Leek et al., 2012) (https://bioconductor.org/packages/release/bioc/html/sva.html). 75% completeness filtering was set as an additional criterion prior to median centering of Cys site intensities and the data was filtered for Cys sites corresponding to a select group of proteins related to muscle protein structure and contraction (File S1) for prioritized analysis.

Statistical analysis was performed using linear regression modeling with the “limma” R package for its enhanced statistical power with low sample size (Smyth, 2005) (https://bioconductor.org/packages/release/bioc/html/limma.html). The models were not stratified by sex due to the limited number of samples and were adjusted for sex, age, and weight of the participants as covariates to test against the phenotypes of leg power, leg strength, walking speed, and cardiopulmonary fitness. Cys sites with statistically significant associations were defined by a *p* < 0.05 threshold. For each phenotype, significantly associated Cys sites were plotted on heatmaps if they had 100% completeness across all samples and Cys oxidation levels for patients without phenotype data were excluded from heatmaps. Heatmap phenotypes were converted to Z‐score and samples correspondingly arranged prior to row clustering. Heatmaps were segregated into 6 distinct clusters defined by the hierarchical clustering dendrogram. Cluster centroids were defined by averaging feature intensities for each sample within a cluster. Cluster centroids were then plotted against non‐Z‐scored phenotype to produce cluster correlation plots scored by Pearson correlation. Cluster‐to‐phenotype correlation was further evaluated by limma using cluster centroids as a combined feature (File S5).

## AUTHOR CONTRIBUTIONS

S.R.C., and W.J.Q. conceived the study. P.M.C. and L.Y.L. selected the samples for this study. N.J.D., M.J.G., J.B.T., I.K.A, and R.J.M performed the proteomics experiments and data collection. S.S.K., and N.J.D. conducted data analysis. L.Y.L., T.A.M., T.J.S., V.A.P. contributed and reviewed statistical analysis. N.J.D., W.J.Q., and S.R.C wrote the manuscript. C.M.D., A.B.N., S.B.K. P.A.K., D.J.M, P.M.C., B.H.G., R.T.H., P.M.Ca., and K.A.E. reviewed and edited the manuscript. S.R.C. and P.M.Ca. are consultants to Bioage Labs. P.M.Ca. is a consultant to and owns stock in MyoCorps.

## FUNDING INFORMATION

The Study of Muscle, Mobility and Aging (SOMMA) is supported by funding from the National Institute on Aging, grant number AG059416. Study infrastructure support was funded in part by NIA Claude D. Pepper Older American Independence Centers at University of Pittsburgh (P30AG024827) and Wake Forest University (P30AG021332) and the Clinical and Translational Science Institutes, funded by the National Center for Advancing Translational Science, at Wake Forest University (UL1 0TR001420). Additional support for this study was provided by the Longevity Consortium (U19AG023122) and the Molecular Transducers of Physical Activity Consortium (U24 DK112349).

## CONFLICT OF INTEREST STATEMENT

All other authors declare no conflict of interest.

## Supporting information


Figures S3‐S10.

File S1.

File S2.

File S3.

File S4.

File S5.

## Data Availability

The mass spectrometry data have been deposited to the MassIVE repository with the dataset accession: MSV000093136. The data will also be available through the ProteomeXchange with the accession number: PXD046200. SOMMA data are also available at https://sommaonline.ucsf.edu/.
