# Supplementary material for: Signatures of cysteine oxidation on muscle structural and contractile proteins are associated with physical performance and muscle function in older adults: Study of Muscle, Mobility and Aging (SOMMA)
Source: Aging Cell. 2024 Feb 8;23(6):e14094. doi: 10.1111/acel.14094 (PMC11166363; doi:10.1111/acel.14094)
Supplement: Supplementary file 1 — Figures S3‐S10. [file ACEL-23-e14094-s005.pdf]

Figure S1

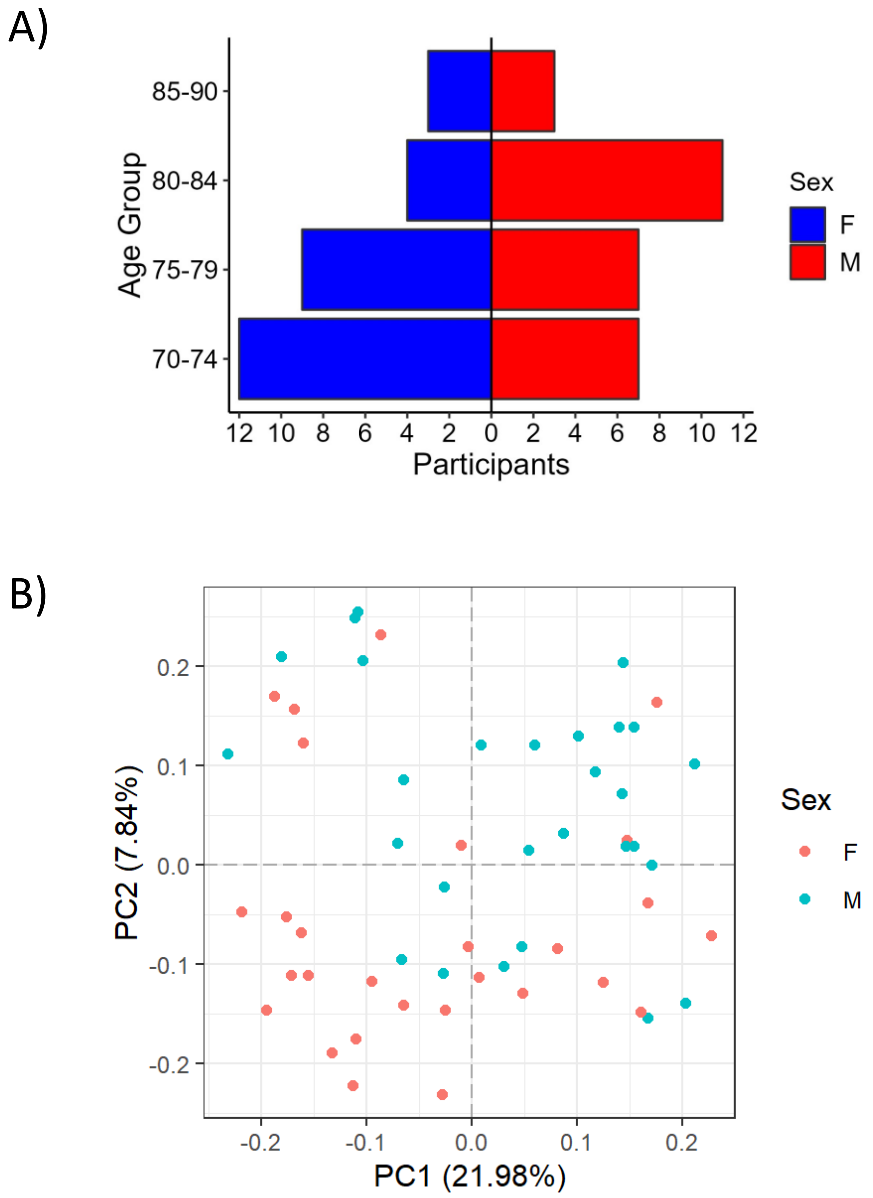

Figure S1. Additional participant characteristics. A) Pyramid plot representing age distribution of the cohort in this study for both sexes. B) Variation in muscle protein oxidation among participants as represented by principal component analysis (PCA) plot of redox proteomics data with each data point (participant) colored by sex.

Figure S2

A)

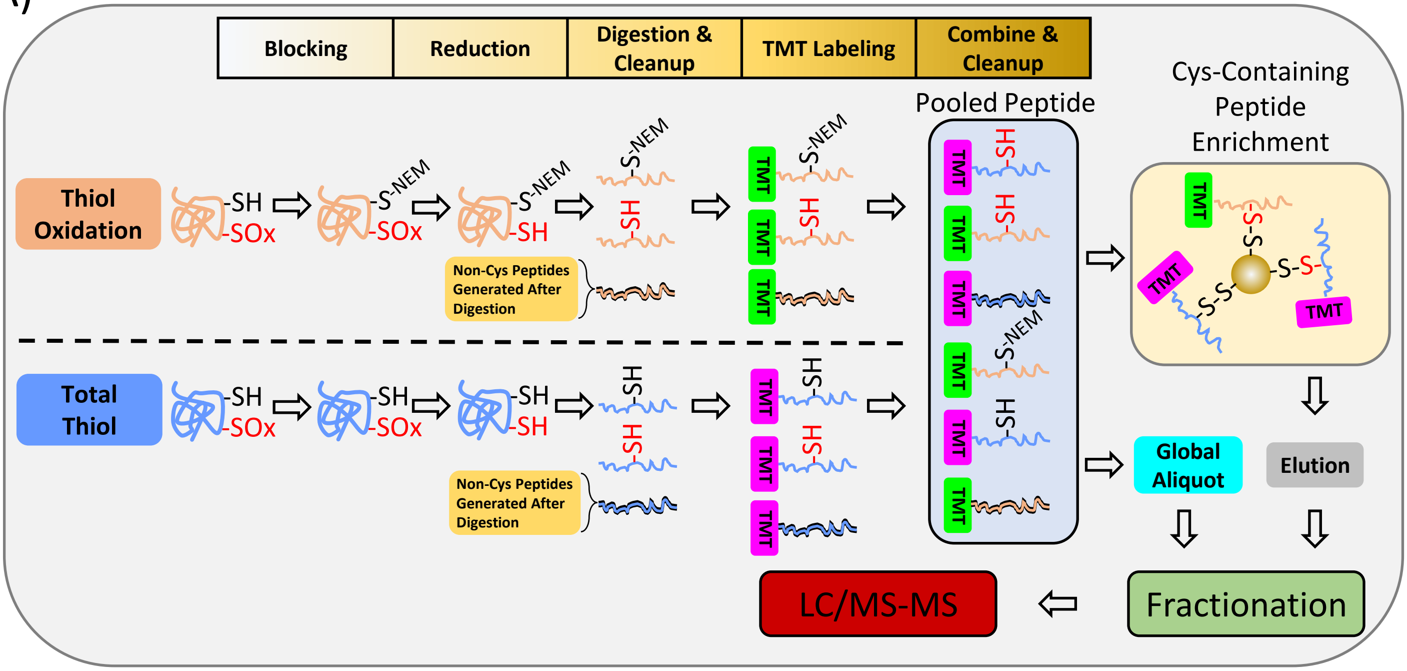

B)

126	127N	127C	128N	128C	129N	129C	130N	130C	131N	131C	132N	132C	133N	134C	135N
Thiol Oxidation (n=14)														Total Thiol (n=2)	

Figure S2. Integrated global and redox proteomics workflow. A) The RAC-TMT workflow (Guo et al., 2014) was modified to integrate both global and redox proteomics workflows into the same processing procedure from the same sample. The blocking and reduction strategies critical for measuring total oxidation of Cys sites were incorporated into a standard global proteomic processing workflow, ultimately allowing for both enrichment of oxidatively modified peptides and global proteomics from the same pool of peptide. The global proteomic data is used to calibrate the redox proteomics data in a similar manner as with phosphoproteomics data, to distinguish changes in Cys oxidation from changes in protein expression. Off-line reverse phase high pressure liquid chromatography fractionation of enriched redox peptides was implemented to increase coverage, as was recently demonstrated in one of our previous reports (Day et al., 2022). Total thiol channels were incorporated for a signal boosting strategy as described in (Day et al., 2022), as well as to provide enough peptide material for offline fractionation. B) Representative TMT18plex design. Muscle biopsy samples were randomly assigned different channels within a multiplex. A total of 4 plexes were generated to analyze 56 samples, where pooled samples were used to generate total thiol samples within each plex.

Figure S3

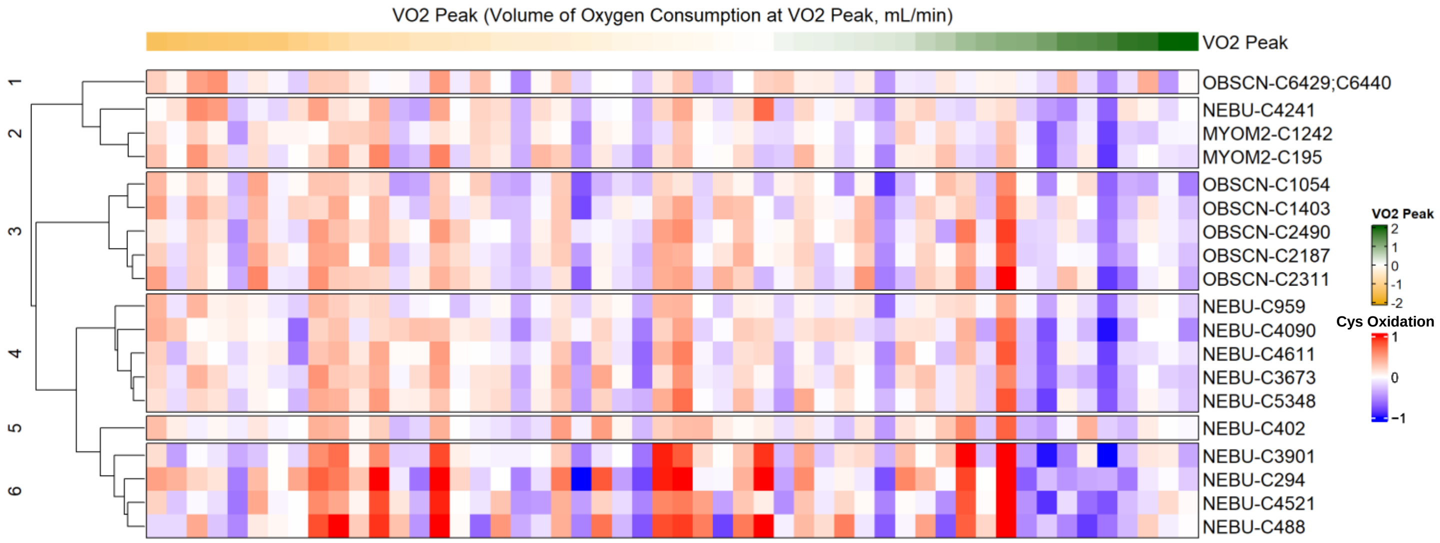

Figures S3-S10. Heatmaps and correlation plots of all Cys sites that are significantly associated with the four phenotypes investigated. For the heatmaps (**Figures S3, S5, S7, S9**), each row represents a Cys site with oxidation levels that are significantly associated with a phenotype, while each column represents a participant. Columns are ranked in ascending order from left to right based on phenotypic measurement (i.e., lower to higher performance), where the phenotypic values are represented by median-centered Z-scores in the row above each heatmap, while Cys oxidation levels were scaled by median-centering. Protein identities are in UniProt format. Sites passing an adjusted p value < 0.05 cutoff are denoted by ‘*’. For each cluster in a heatmap, Cys sites were plotted for correlation analysis (**Figures S4, S6, S8, S10**), where the mean level of oxidation across all Cys sites were plotted for each participant against their phenotypic measurement. *R* and p values are derived from Pearson correlation.

Figure S4

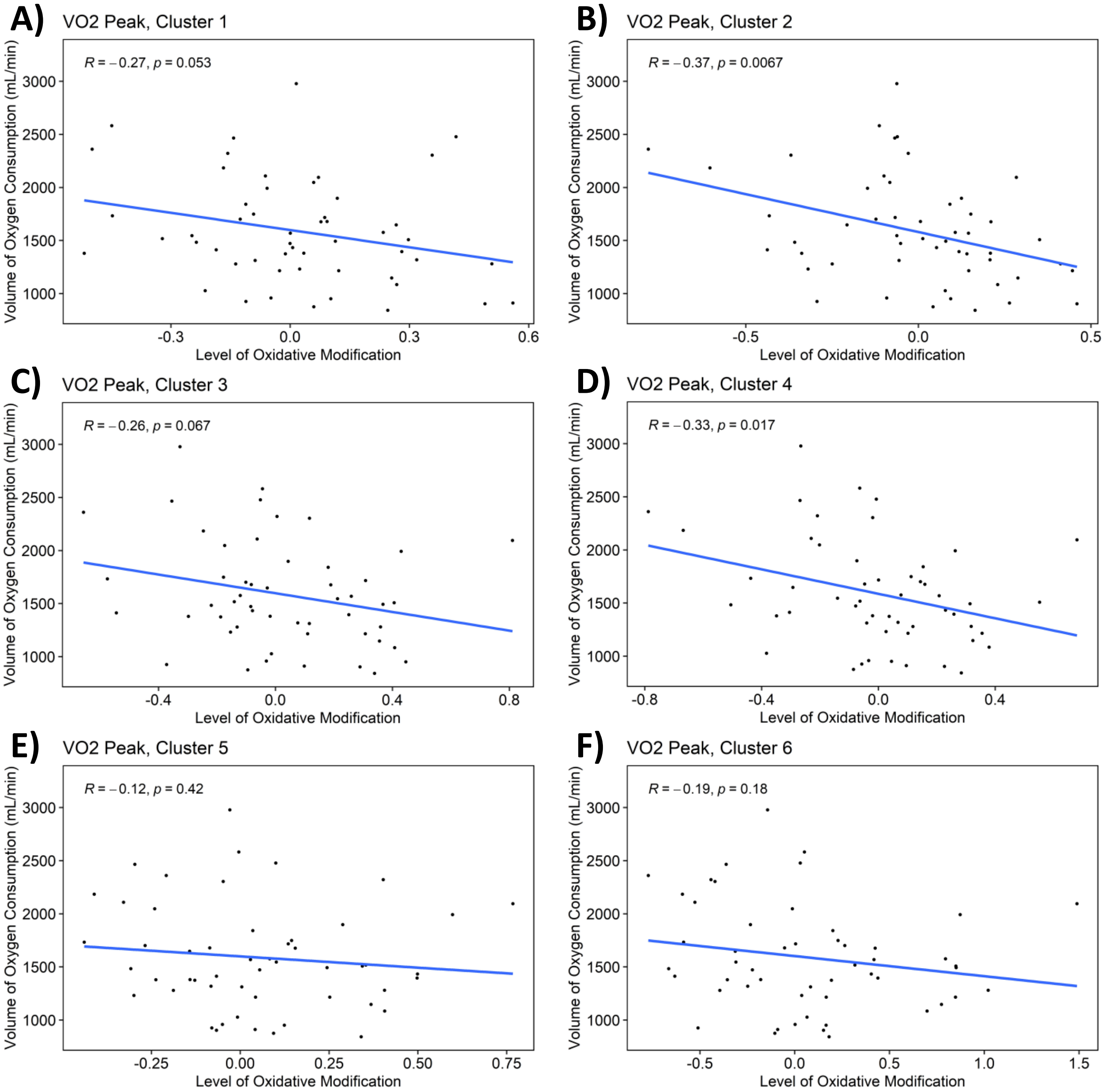

A)
VO2 Peak, Cluster 1
R = −0.27, p = 0.053
B)
VO2 Peak, Cluster 2
R = −0.37, p = 0.0067
C)
VO2 Peak, Cluster 3
R = −0.26, p = 0.067
D)
VO2 Peak, Cluster 4
R = −0.33, p = 0.017
E)
VO2 Peak, Cluster 5
R = −0.12, p = 0.42
F)
VO2 Peak, Cluster 6
R = −0.19, p = 0.18
Volume of Oxygen Consumption (mL/min)
Level of Oxidative Modification

Figure S5

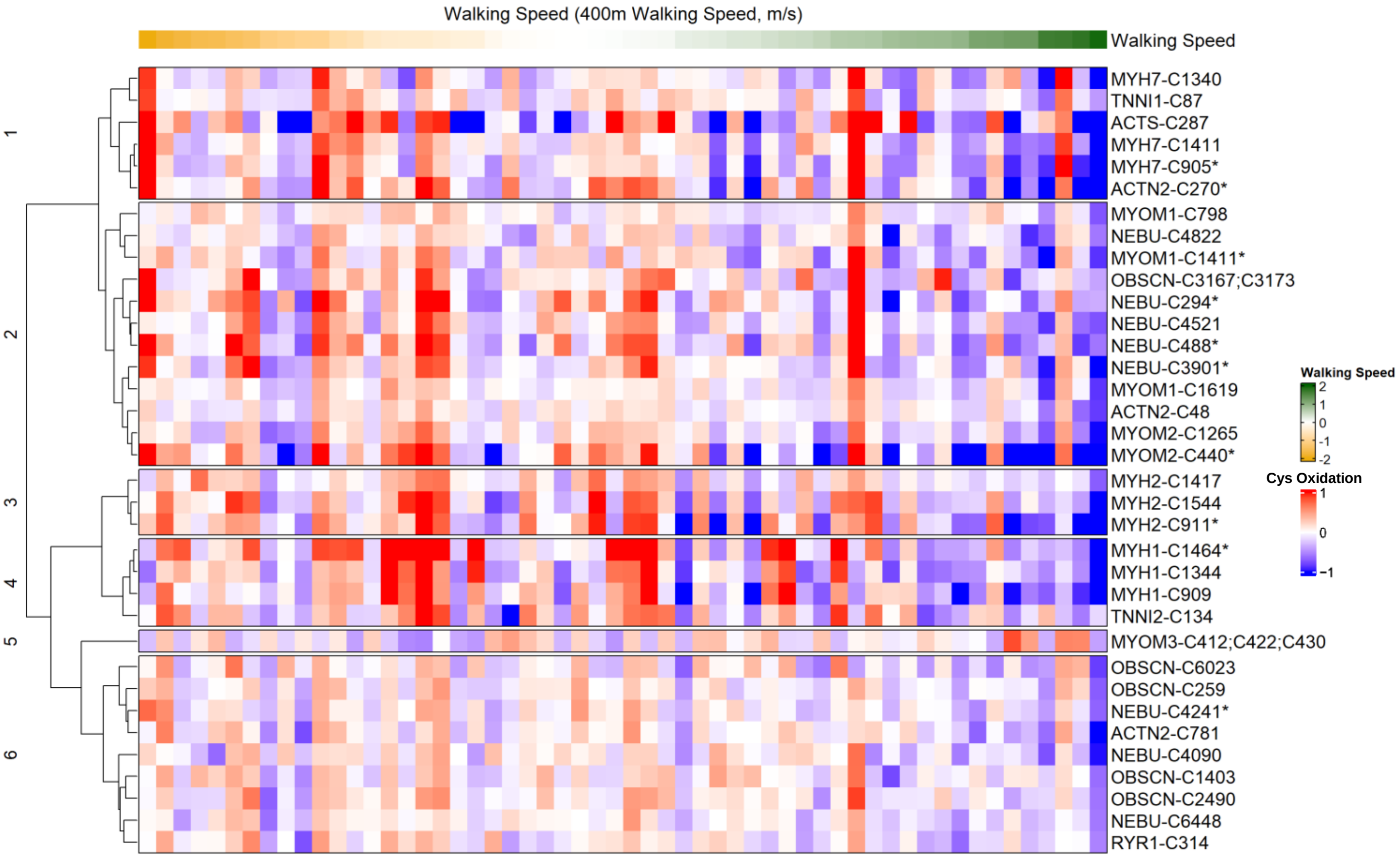
Walking Speed (400m Walking Speed, m/s)
Walking Speed
1
2
3
4
5
6
MYH7-C1340
TNNI1-C87
ACTS-C287
MYH7-C1411
MYH7-C905*
ACTN2-C270*
MYOM1-C798
NEBU-C4822
MYOM1-C1411*
OBSCN-C3167;C3173
NEBU-C294*
NEBU-C4521
NEBU-C488*
NEBU-C3901*
MYOM1-C1619
ACTN2-C48
MYOM2-C1265
MYOM2-C440*
MYH2-C1417
MYH2-C1544
MYH2-C911*
MYH1-C1464*
MYH1-C1344
MYH1-C909
TNNI2-C134
MYOM3-C412;C422;C430
OBSCN-C6023
OBSCN-C259
NEBU-C4241*
ACTN2-C781
NEBU-C4090
OBSCN-C1403
OBSCN-C2490
NEBU-C6448
RYR1-C314
Walking Speed
2
1
0
-1
-2
Cys Oxidation
1
0
−1

Figure S6

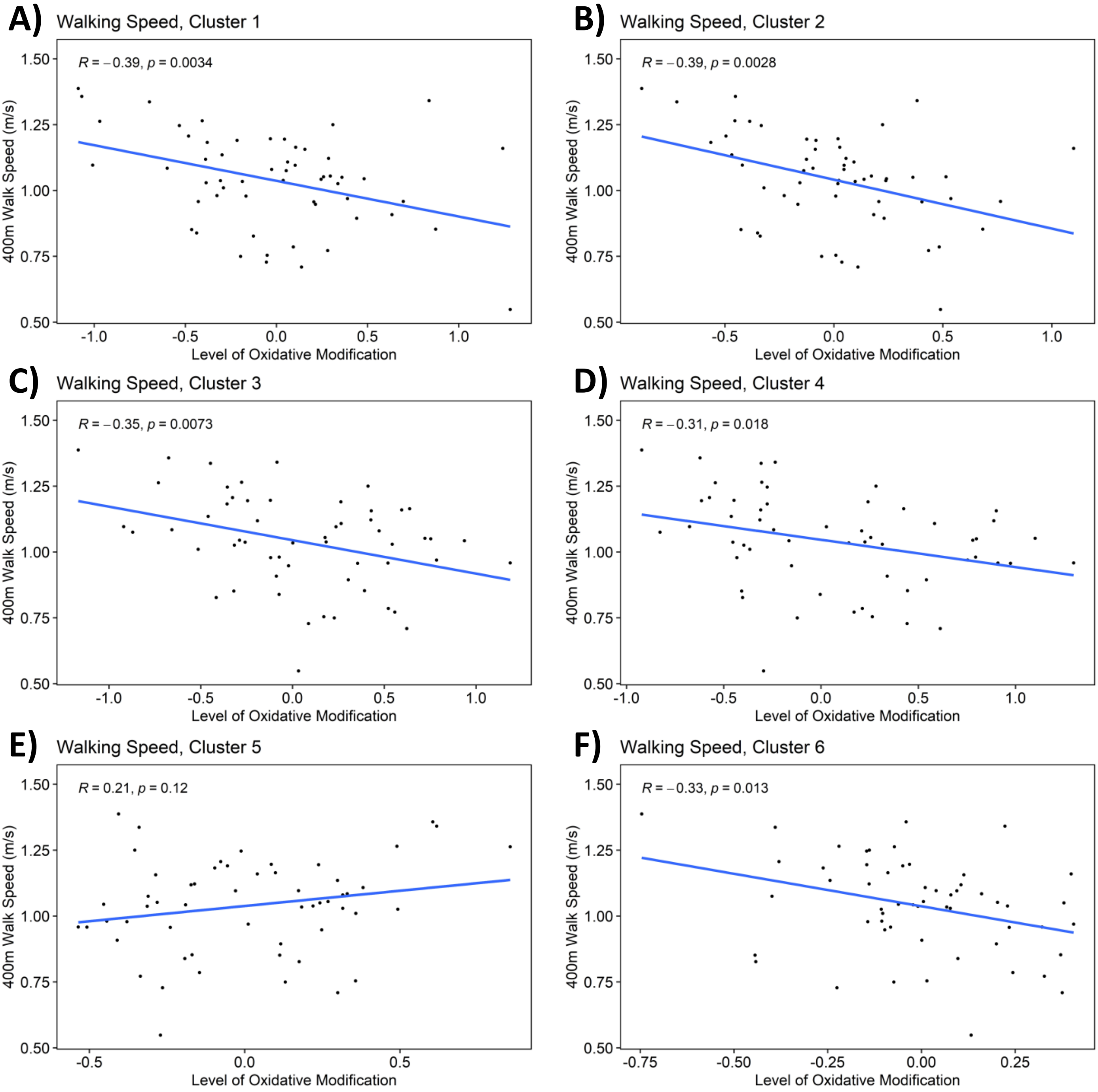
A)
Walking Speed, Cluster 1
R = –0.39, p = 0.0034
B)
Walking Speed, Cluster 2
R = –0.39, p = 0.0028
C)
Walking Speed, Cluster 3
R = –0.35, p = 0.0073
D)
Walking Speed, Cluster 4
R = –0.31, p = 0.018
E)
Walking Speed, Cluster 5
R = 0.21, p = 0.12
F)
Walking Speed, Cluster 6
R = –0.33, p = 0.013
400m Walk Speed (m/s)
Level of Oxidative Modification

Figure S7

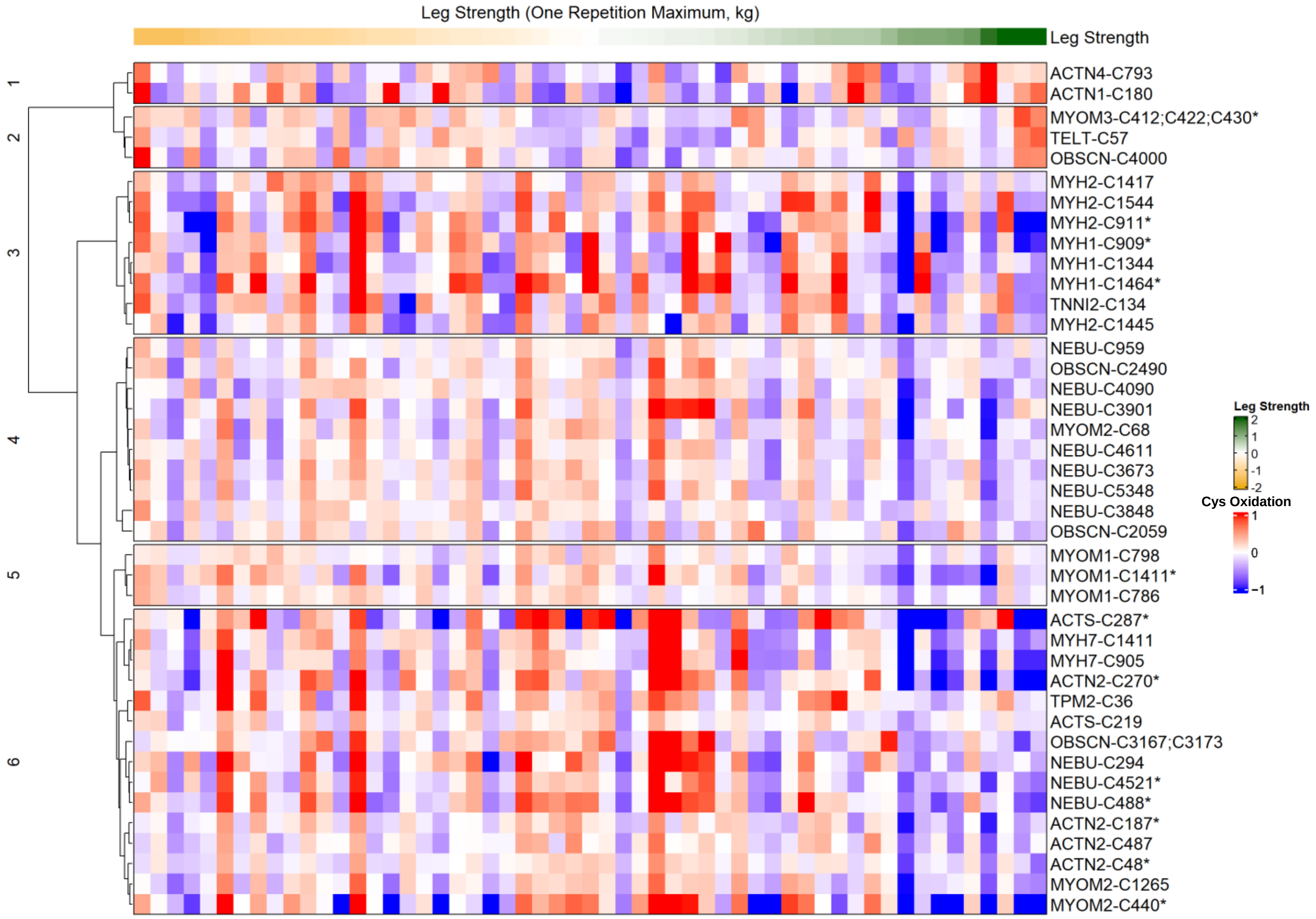

Leg Strength (One Repetition Maximum, kg)
Leg Strength
1
2
3
4
5
6
ACTN4-C793
ACTN1-C180
MYOM3-C412;C422;C430*
TELT-C57
OBSCN-C4000
MYH2-C1417
MYH2-C1544
MYH2-C911*
MYH1-C909*
MYH1-C1344
MYH1-C1464*
TNNI2-C134
MYH2-C1445
NEBU-C959
OBSCN-C2490
NEBU-C4090
NEBU-C3901
MYOM2-C68
NEBU-C4611
NEBU-C3673
NEBU-C5348
NEBU-C3848
OBSCN-C2059
MYOM1-C798
MYOM1-C1411*
MYOM1-C786
ACTS-C287*
MYH7-C1411
MYH7-C905
ACTN2-C270*
TPM2-C36
ACTS-C219
OBSCN-C3167;C3173
NEBU-C294
NEBU-C4521*
NEBU-C488*
ACTN2-C187*
ACTN2-C487
ACTN2-C48*
MYOM2-C1265
MYOM2-C440*
Leg Strength
2
1
0
-1
-2
Cys Oxidation
1
0
−1

Figure S8

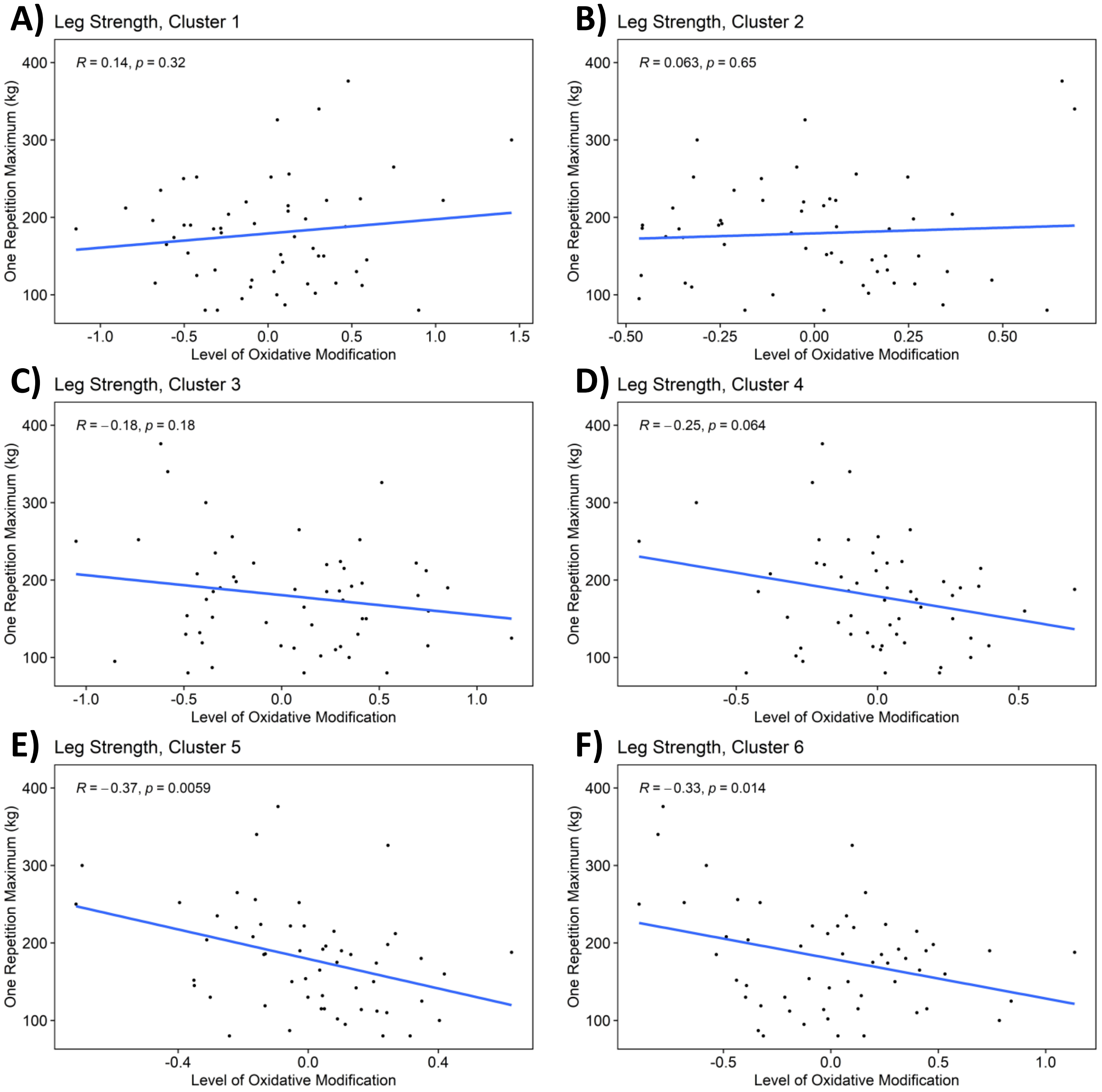

A) Leg Strength, Cluster 1
R = 0.14, p = 0.32
B) Leg Strength, Cluster 2
R = 0.063, p = 0.65
C) Leg Strength, Cluster 3
R = −0.18, p = 0.18
D) Leg Strength, Cluster 4
R = −0.25, p = 0.064
E) Leg Strength, Cluster 5
R = −0.37, p = 0.0059
F) Leg Strength, Cluster 6
R = −0.33, p = 0.014
One Repetition Maximum (kg)
Level of Oxidative Modification
100
200
300
400
-1.0
-0.5
0.0
0.5
1.0
1.5
-0.50
-0.25
0.00
0.25
0.50
-0.4
0.4

Figure S9

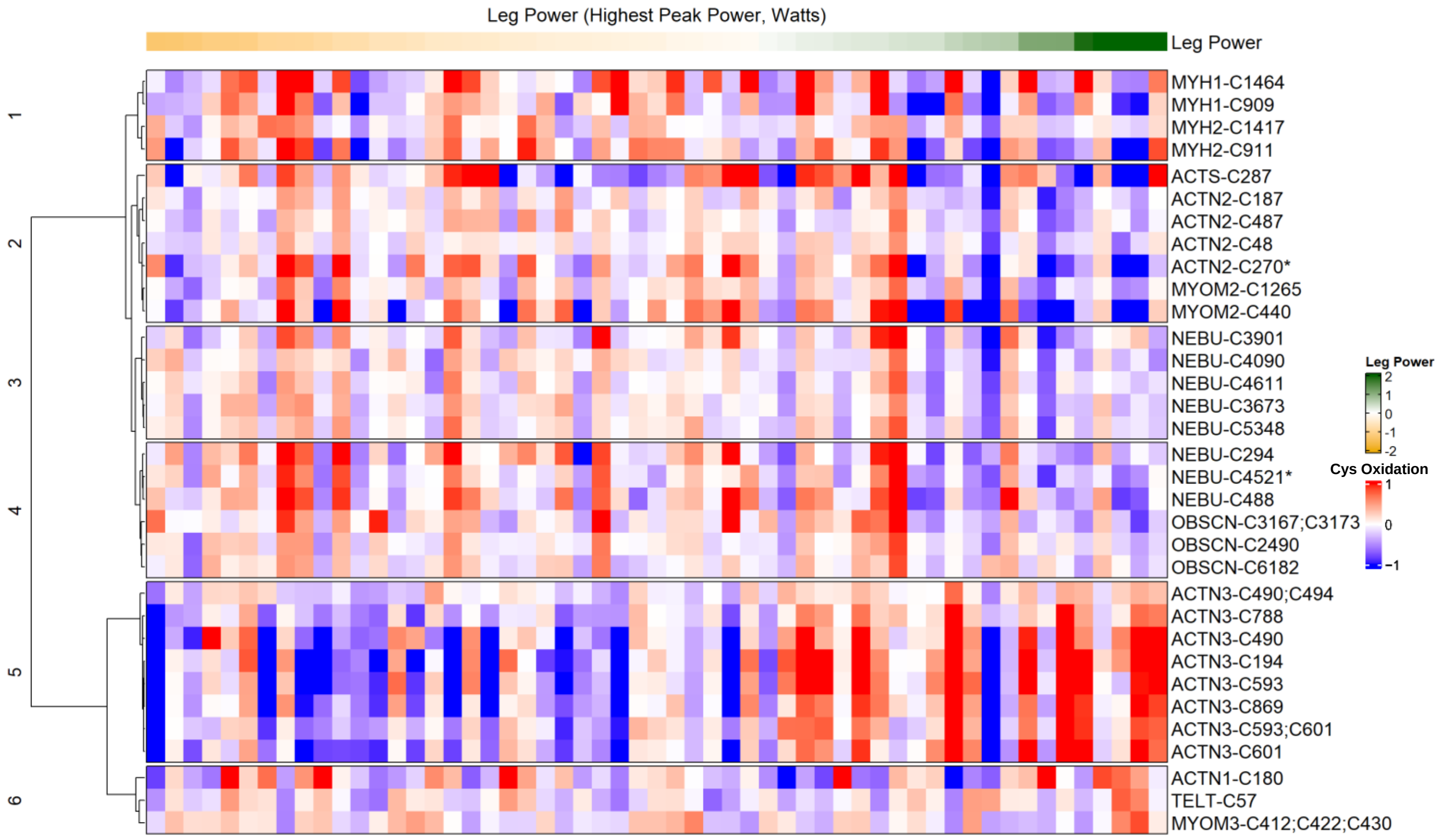

Leg Power (Highest Peak Power, Watts)
Leg Power
1
2
3
4
5
6
MYH1-C1464
MYH1-C909
MYH2-C1417
MYH2-C911
ACTS-C287
ACTN2-C187
ACTN2-C487
ACTN2-C48
ACTN2-C270*
MYOM2-C1265
MYOM2-C440
NEBU-C3901
NEBU-C4090
NEBU-C4611
NEBU-C3673
NEBU-C5348
NEBU-C294
NEBU-C4521*
NEBU-C488
OBSCN-C3167;C3173
OBSCN-C2490
OBSCN-C6182
ACTN3-C490;C494
ACTN3-C788
ACTN3-C490
ACTN3-C194
ACTN3-C593
ACTN3-C869
ACTN3-C593;C601
ACTN3-C601
ACTN1-C180
TELT-C57
MYOM3-C412;C422;C430
Leg Power
2
1
0
-1
-2
Cys Oxidation
1
0
-1

Figure S10

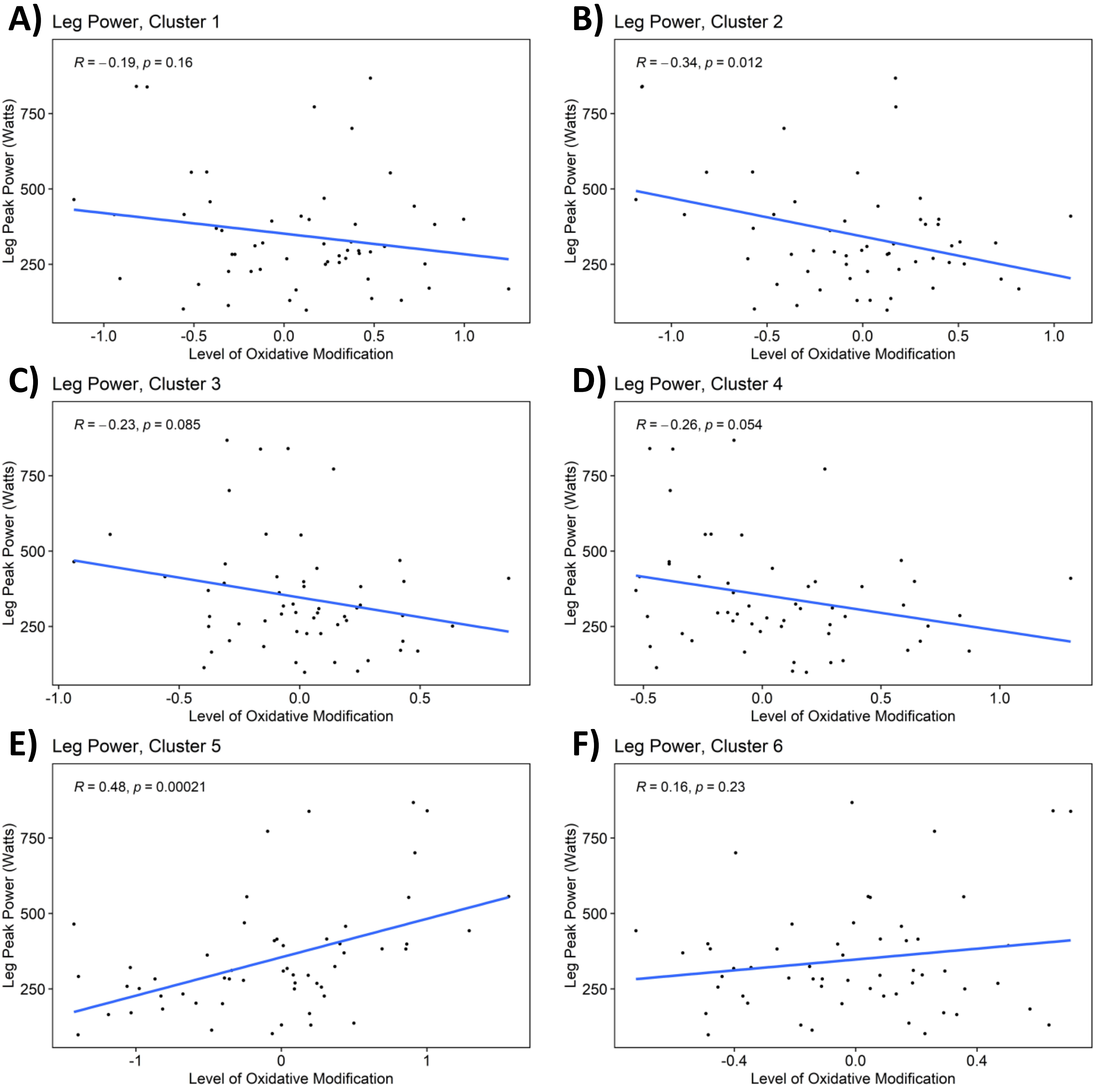

A) Leg Power, Cluster 1
R = −0.19, p = 0.16
B) Leg Power, Cluster 2
R = −0.34, p = 0.012
C) Leg Power, Cluster 3
R = −0.23, p = 0.085
D) Leg Power, Cluster 4
R = −0.26, p = 0.054
E) Leg Power, Cluster 5
R = 0.48, p = 0.00021
F) Leg Power, Cluster 6
R = 0.16, p = 0.23
Leg Peak Power (Watts)
Level of Oxidative Modification
