# Supplementary material for: Signatures of cysteine oxidation on muscle structural and contractile proteins are associated with physical performance and muscle function in older adults: Study of Muscle, Mobility and Aging (SOMMA)
Source: Aging Cell. 2024 Feb 8;23(6):e14094. doi: 10.1111/acel.14094 (PMC11166363; doi:10.1111/acel.14094)
Supplement: Supplementary file 2 — File S1. [file ACEL-23-e14094-s001.docx]

Supplemental File S1: Muscle proteins investigated in this study.

| Protein | UniProt ID | UniProt name | Gene Name | Location in Sarcomere |
| --- | --- | --- | --- | --- |
| Alpha-actinin-1 | [P12814](https://www.uniprot.org/uniprotkb/P12814/entry) | ACTN1_HUMAN | ACTN1 | Z-line |
| Alpha-actinin-2 | [P35609](https://www.uniprot.org/uniprotkb/P35609/entry) | ACTN2_HUMAN | ACTN2 | Z-line |
| Alpha-actinin-3 | [Q08043](https://www.uniprot.org/uniprotkb/Q08043/entry) | ACTN3_HUMAN | ACTN3 | Z-line |
| Alpha-actinin-4 | [O43707](https://www.uniprot.org/uniprotkb/O43707/entry) | ACTN4_HUMAN | ACTN4 | Z-line |
| Myosin light chain 3 | [P08590](https://www.uniprot.org/uniprotkb/P08590/entry) | MYL3_HUMAN | MYL3 | Thick filament |
| Myosin-1 | [P12882](https://www.uniprot.org/uniprotkb/P12882/entry) | MYH1_HUMAN | MYH1 | Thick filament |
| Myosin-2 | [Q9UKX2](https://www.uniprot.org/uniprotkb/Q9UKX2/entry) | MYH2_HUMAN | MYH2 | Thick filament |
| Myosin-7 | [P12883](https://www.uniprot.org/uniprotkb/P12883/entry) | MYH7_HUMAN | MYH7 | Thick filament |
| Troponin C, skeletal muscle | [P02585](https://www.uniprot.org/uniprotkb/P02585/entry) | TNNC2_HUMAN | TNNC2 | Thin filament |
| Troponin I, slow skeletal muscle (Troponin I, slow-twitch isoform) | [P19237](https://www.uniprot.org/uniprotkb/P19237/entry) | TNNI1_HUMAN | TNNI1 | Thin filament |
| Troponin I, fast skeletal muscle (Troponin I, fast-twitch isoform) | [P48788](https://www.uniprot.org/uniprotkb/P48788/entry) | TNNI2_HUMAN | TNNI2 | Thin filament |
| Troponin C, slow skeletal and cardiac muscles (TN-C) | [P63316](https://www.uniprot.org/uniprotkb/P63316/entry) | TNNC1_HUMAN | TNNC1 | Thin filament |
| Tropomyosin beta chain | [P07951](https://www.uniprot.org/uniprotkb/P07951/entry) | TPM2_HUMAN | TPM2 | Thin filament |
| Tropomyosin alpha-1 chain | [P09493](https://www.uniprot.org/uniprotkb/P09493/entry) | TPM1_HUMAN | TPM1 | Thin filament |
| Actin, alpha skeletal muscle | [P68133](https://www.uniprot.org/uniprotkb/P68133/entry) | ACTS_HUMAN | ACTA1 | Thin filament |
| Myomesin-1 | [P52179](https://www.uniprot.org/uniprotkb/P52179/entry) | MYOM1_HUMAN | MYOM1 | M-line |
| Myomesin-2 | [P54296](https://www.uniprot.org/uniprotkb/P54296/entry) | MYOM2_HUMAN | MYOM2 | M-line |
| Myomesin-3 | [Q5VTT5](https://www.uniprot.org/uniprotkb/Q5VTT5/entry) | MYOM3_HUMAN | MYOM3 | M-line |
| Telethonin | [O15273](https://www.uniprot.org/uniprotkb/O15273/entry) | TELT_HUMAN | TCAP | Z-line |
| Obscurin | [Q5VST9](https://www.uniprot.org/uniprotkb/Q5VST9/entry) | OBSCN_HUMAN | OBSCN | M-line |
| Nebulin | [P20929](https://www.uniprot.org/uniprotkb/P20929/entry) | NEBU_HUMAN | NEB | Thin filament |
| Ryanodine receptor 1 | [P21817](https://www.uniprot.org/uniprotkb/P21817/entry) | RYR1_HUMAN | RYR1 | External of sarcomere |
